# Denitrification genotypes of endospore-forming *Bacillota*

**DOI:** 10.1093/ismeco/ycae107

**Published:** 2024-09-04

**Authors:** Emma Bell, Jianwei Chen, William D L Richardson, Milovan Fustic, Casey R J Hubert

**Affiliations:** Department of Biological Sciences, University of Calgary, 2500 University Drive NW, Calgary, Alberta T2N 1N4, Canada; Department of Biological Sciences, University of Calgary, 2500 University Drive NW, Calgary, Alberta T2N 1N4, Canada; Department of Biological Sciences, University of Calgary, 2500 University Drive NW, Calgary, Alberta T2N 1N4, Canada; Department of Biological Sciences, University of Calgary, 2500 University Drive NW, Calgary, Alberta T2N 1N4, Canada; Department of Geology, Nazarbayev University, 53 Kabanbay Batyr Ave, Astana 010000, Kazakhstan; Department of Biological Sciences, University of Calgary, 2500 University Drive NW, Calgary, Alberta T2N 1N4, Canada

**Keywords:** denitrification, metagenomics, endospores, Bacillota, nitrate

## Abstract

Denitrification is a key metabolic process in the global nitrogen cycle and is performed by taxonomically diverse microorganisms. Despite the widespread importance of this metabolism, challenges remain in identifying denitrifying populations and predicting their metabolic end-products based on their genotype. Here, genome-resolved metagenomics was used to explore the denitrification genotype of *Bacillota* enriched in nitrate-amended high temperature incubations with confirmed N_2_O and N_2_ production. A set of 12 hidden Markov models (HMMs) was created to target the diversity of denitrification genes in members of the phylum *Bacillota*. Genomic potential for complete denitrification was found in five metagenome-assembled genomes from nitrate-amended enrichments, including two novel members of the *Brevibacillaceae* family. Genomes of complete denitrifiers encode N_2_O reductase gene clusters with clade II-type *nosZ* and often include multiple variants of the nitric oxide reductase gene*.* The HMM set applied to all genomes of *Bacillota* from the Genome Taxonomy Database identified 17 genera inferred to contain complete denitrifiers based on their gene content. Among complete denitrifiers it was common for three distinct nitric oxide reductases to be present (qNOR, bNOR, and sNOR) that may reflect the metabolic adaptability of *Bacillota* in environments with variable redox conditions.

## Introduction

Denitrification is a key metabolic process in the nitrogen cycle featuring sequential reduction of nitrate to nitrite and then gaseous metabolites (NO_3_^−^ → NO_2_^−^ → NO → N_2_O → N_2_). Different enzymes catalyze each of the four reduction reactions such that this modular metabolism can be performed by a single microorganism or a microbial consortium. When performed modularly, microorganisms can achieve complete denitrification by cross-feeding intermediates [1]. If denitrification is incomplete, this can give rise to the release of the greenhouse gas N_2_O to the atmosphere [2, 3]. Denitrification is prevalent in terrestrial and aquatic environments where oxic and anoxic conditions occur close to each other [4]. In soil environments, denitrification can contribute to losses of fixed nitrogen to the atmosphere reducing soil fertility and plant yield [5]. On the other hand, denitrification in wetlands mitigates the transport of nitrogen from land to lakes and coastal waters where excess nitrogen can cause eutrophication [6]. Nitrogen removal in wastewater and agricultural sectors via denitrification similarly represents a critical step that limits the release of excess to nitrogen into watersheds [7, 8]. Identifying microorganisms contributing towards denitrification in different environments is therefore of ecological and industrial importance.

Microorganisms known to perform denitrification are taxonomically diverse and span both bacterial and archaeal domains [9, 10]. The taxonomic diversity of denitrifiers means that this metabolism cannot be easily linked to phylogeny [11]. Instead, characterization of denitrifying populations relies on the presence of metabolic genes for each of the reduction steps. Nitrate reduction to nitrite (NO_3_^−^ → NO_2_^−^) is catalyzed by the membrane-bound Nar enzyme or periplasmic Nap enzyme, both of which can be found in denitrifiers and nitrate-ammonifiers (NO_3_^−^ → NH_4_^+^) [12, 13]. Thus, the presence of nitrite reductase (Nir) genes for nitrite reduction to nitric oxide (NO_2_^−^ → NO), nitric oxide reductase (Nor) genes for nitric oxide reduction to nitrous oxide (NO → N_2_O), and the nitrous oxide reductase (Nos) gene for nitrous oxide reduction to dinitrogen (N_2_O → N_2_) differentiates the denitrification pathway from dissimilatory nitrate reduction to ammonium (DNRA).

Enzymes from the denitrification pathway exhibit broad taxonomic and sequence diversity. Nitrite reduction to nitric oxide is catalyzed by two structurally different enzymes, Cu-type NirK and cytochrome cd-1 type NirS, that have different evolutionary histories [14–16]. Nitric oxide reductases are members of the heme-copper oxidase (HCO) superfamily and are ancestral to terminal oxidases for aerobic respiration [17, 18]. Four Nor enzyme families have been biochemically characterized: cNOR [19], qNOR [20], bNOR (formerly Cu_A_NOR; [21]) and eNOR [22]. Cytochrome *c*-dependent (cNOR) and quinol-dependent (qNOR) Nor enzymes are related to C-family oxygen reductases whereas bNOR and the recently characterized eNOR are related to B-family oxygen reductases [18, 22]. Based on phylogenomic analysis and conserved proton channels, an additional three enzymes related to B-family oxygen reductases, sNOR, gNOR, and nNOR, have also been proposed [22].

In contrast to nitric oxide reduction, nitrous oxide reduction is catalyzed by just one enzyme, NosZ. This enzyme, however, forms two distinct groups known as clade I (typical) or clade II (atypical) characterized by different secretionary pathways (tat and sec, respectively) [10, 23]. Clade I is represented by well-studied denitrifying *Proteobacteria* (alpha-, beta-, and gamma-) whereas clade II is taxonomically diverse and encompasses at least 12 bacterial and archaeal phyla (subclades A-K) [10, 24].

The broad diversity and sequence divergence among denitrification enzymes gives rise to well-documented coverage limitations for PCR primers and probes [10, 11, 15, 25, 26]. This makes it challenging to accurately estimate the diversity and abundance of denitrification genes. Metagenomics circumvents primer bias limitations and is therefore advantageous for studying denitrification. Here, genome-resolved metagenomics was employed to explore the gene content of microorganisms enriched in the presence of nitrate in heated oil sands from outcrops in Alberta, Canada. Understanding thermophilic populations in oil sands and their nitrate-reducing metabolism is of interest for technologies that target in situ microbial activity [27]. Compared to conventional crude oil reservoir ecosystems, oil sands are not well characterized microbiologically but the presence of both mesophilic and thermophilic populations in riverbank outcrops and subsurface deposits has been reported [28, 29]. Our data show that dormant thermophilic endospore-forming (thermospore) populations with distinct denitrification genotypes are present in the oil sands microbiome.

## Materials and Methods

### Sample collection

Samples were collected from the Athabasca oil sands in Alberta, Canada, in June 2019. In this region, oil sands are present at various depths (up to 100 s of meters) and outcrops are naturally exposed along the riverbanks of the Athabasca River and its tributaries. Oil sands samples were collected from an outcrop at the Hangingstone River (56°42′38”N, 111°23′51″) in Fort McMurray. Samples were stored in a cold room at 4°C until incubations were established. Parallel samples were frozen for DNA extraction and analyzed to represent unincubated samples.

### Nitrate-amended enrichments

Approximately 30 g oil sands inoculum containing ~14% bitumen, 4% water, and 82% sand was combined with 60 mL anoxic medium in 160 mL Wheaton glass serum bottles. Growth medium was based upon media previously used to isolate nitrate-reducing and fermentative thermophilic *Bacillota* (formerly *Firmicutes*) from hydrocarbon environments (Adkins et al., 1992; Salinas et al., 2004) and contained (L^−1^ distilled water): 0.2 g MgCl_2_•6H_2_O, 0.1 g KCl, 1 g NH_4_Cl, 0.1 g CaCl_2_•2H_2_O, 0.3 g K_2_HPO_4_, 0.3 g KH_2_PO_4_, 1 g NaCl, 0.2 g yeast extract. NaNO_3_ (2 g/L) was added as an electron acceptor to promote denitrification. Oil sands are heavily biodegraded such that microbial growth with bitumen as the sole carbon and electron source in these microcosms is negligible [30, 31]. Glucose (0.9 g/L) was therefore added to the enrichments as an easily biodegradable organic substrate. Cysteine hydrochloride (0.5 g/L), NaHCO_3_ (2.5 g/L), vitamins and trace minerals were added from sterile stock solutions. Anoxia was established with He to enable sub-samples of headspace gas to be analyzed for the presence of nitrogen compounds. Throughout the incubation period, sub-samples of the sand and water mixture (1.5 mL) were periodically removed from enrichments using a He-flushed syringe. Sub-samples were centrifuged (10 000 × g for 5 minutes) with supernatants filtered (0.2 μm) and frozen eventual for chemical analysis and pellets frozen for eventual DNA extraction.

### Headspace gas measurement

Headspace gases (1 mL) were extracted from experimental incubations with a He-flushed syringe and immediately injected into two chain-connected sample loops on an Agilent 7890B gas chromatograph (GC). CO_2_ was first separated on a Hayesep N packing column (stainless steel tubing, 0.5 m length, 1/8″ OD, 2 mm ID, mesh size 80/100) followed by N_2_ separation on a MolSieve 5A packing column (UltiMetal tubing, 2.44 m length, 1/8″ OD, 2 mm ID, mesh size 60/80) with He carrier gas. Both CO_2_ and N_2_ were measured by thermal conductivity detector at 200°C. Through a second line, N_2_O was separated on a Hayseed Q packing column (stainless steel tubing, 6′ length, 1/8″ OD, 2.1 mm ID, mesh size 80/200) with Ar/CH_4_ 5/95% carrier gas. N_2_O was measured by electron capture detector at 300°C. All columns were set in the same oven with a working temperature of 105°C.

### Chemical analyses

Nitrate and nitrite were measured with a Dionex ICS-5000 reagent-free ion chromatography system equipped with an anion-exchange column (Dionex IonPac AS22; 4 × 250 mm). The eluent was 4.5 mM K_2_CO_3_/1.4 mM KHCO_3_, the flow rate was 1.3 mL/min, and the column temperature was 30°C. Organic acids (formate, acetate, propionate, lactate, butyrate, and succinate) were measured using UV (210 nm) on an HPLC RSLC Ultimate 3000 equipped with an Aminex HPX-87H, 7.8 × 300 mm analytical column. The isocratic eluent was 5 mM H_2_SO_4_, the flow rate was 0.6 mL/min, and the column oven was heated to 60°C. Glucose was measured by mixing samples with the Glucose (HK) Assay reagent (Sigma-Aldrich) following the manufacturer’s instructions and absorbance was measured on a spectrophotometer at 410 nm.

### DNA extraction

DNA was extracted from frozen pellets (0.25 g) using the Qiagen DNeasy PowerLyzer PowerSoil kit according to the manufacturer’s protocol. DNA concentrations were measured using the dsDNA High Sensitivity assay kit on a Qubit 2.0 fluorometer. DNA yields from heated oil sands ranged between 329–7890 ng DNA g^−1^ oil sand. To represent unincubated samples, DNA was extracted from 8-10 g oil sands (i.e., samples that were frozen when original outcrop samples were collected) using the Qiagen DNeasy PowerMax Soil kit according to the manufacturer’s protocol. Triplicate DNA extractions from these oil sands yielded 172–228 ng DNA g^−1^.

### 16S rRNA gene amplicon sequencing

Amplicon sequencing of the 16S rRNA gene (V4-V5 region) was performed using the bacterial primer set 515F and 926R [32]. Triplicate PCR reactions were pooled then purified using the NucleoMag NGS clean-up and size select kit. Purified PCR products were indexed following Illumina’s 16S rRNA amplicon preparation instructions. Indexed amplicons were verified on an Agilent 2100 Bioanalyzer system and sequenced on a MiSeq benchtop sequencer (Illumina) using the v3 600-cycle (paired end) reagent kit. Primers were trimmed from sequence reads with Cutadapt v4.4 [33] and processed in DADA2 [34] following the recommended pipeline (https://benjjneb.github.io/dada2/tutorial.html). Taxonomy was assigned to amplicon sequence variants (ASVs) with “assignTaxonomy” in DADA2 using the Swedish Biodiversity Infrastructure Sativa curated 16S GTDB database from release R07-RS207 (https://doi.org/10.17044/scilifelab.14869077).

### Metagenome sequencing, read processing, and binning

Metagenomic sequencing was performed on a NovaSeq 6000 (Illumina) with a S4 300 cycle flow cell. Libraries were prepared by shearing to an insert size of 200 bp using a Covaris instrument followed by library construction with the NEB Ultra II DNA library prep kit. Adaptors and low-quality reads were removed with Cutadapt v.1.18 [33] using the wrapper Trimgalore v0.6.7 [35]. Reads from each sample were assembled individually with Megahit v1.2.9 [36] using the “—meta-sensitive” option. Read and assembly statistics are provided in Table S1. Reads were cross-mapped to the assembled contigs with BBMap v38.95 [37] to generate coverage profiles for binning. Contigs from each assembly were then binned with MetaBAT2 [38] and CONCOCT [39] and refined with DAS Tool [40]. Bin completeness and contamination were calculated with CheckM2 [41] and bins with >50% completeness and <10% contamination were retained. Small subunit (SSU) rRNA gene sequences in redundant bins were identified with Metaxa2 v2.2.3 [42]. ASVs from amplicon sequencing were compared to SSU rRNA gene sequences in metagenome-assembled genomes (MAGs) with BLASTn 2.6.0+ [43]. Redundant bins were dereplicated with dRep [44] resulting in 17 non-redundant MAGs (Table S2). Relative abundance of non-redundant MAGs was determined with CoverM using default parameters for “coverm genome” [45].

### Annotation and evaluation MAGs

MAGs were taxonomically classified with Genome Taxonomy Database Toolkit (GTDB-tk) v2.3.2 with reference data R214 [46]. Average amino acid identity (AAI) comparisons between MAGs without close relatives in GTDB (<70% AAI) were determined with AAI calculator [47]. Functional annotation with KEGG and EggNOG databases was performed with GhostKOALA [48] and eggnog-mapper v2.1.7 [49]. Optimal growth temperature was predicted from protein sequences with “tome predOGT” [50] (Table S2) and MAGs were checked for functional and regulatory genes involved in endospore formation (Table S3). MAGs of mesophilic non-endosporulating bacteria included *Actinobacteriota* (×1), *Patescibacteria* (×2), and *Proteobacteria* (×2). These MAGs binned from unheated oil sands inoculum and had low relative abundance in heated samples so were excluded from further analysis. The final non-redundant set of thermospore MAGs contained 12 high quality *Bacillota* genomes (Table S2).

### Annotation of the Nos gene cluster

Genes identified as *nosZ* were checked for the presence of Sec/SPI signal peptides, characteristic of all clade II *nosZ*, with SignalP 6.0 [51]. Genes on the same contig as *nosZ* were then checked for transmembrane helices with DeepTMHMM [52] to identify NosB which contains 4 or 6 transmembrane helices and is typically situated adjacent to NosZ in clade II-type *nosZ* microorganisms [53]. A cytochrome *c* preceding *nosZ* was identified with eggNOG and was determined to be *nosC* [54]. Cellular localization of denitrification genes was predicted with PSORTb. [55]. Nos gene clusters were visualized with the gggenes extension [56] for gglot2 [57].

### Hidden Markov models for denitrification

A denitrification gene set was compiled from both custom hidden Markov models (HMMs) and TIGRfams (Table S4). Six HMMs were created for *nirK*, *nirS*, *qnor*, *bnor*, *snor*, and *nosB.* Amino acid sequences from genes of interest were retrieved from studies biochemically characterizing and describing the enzymes [15, 20, 22, 58, 59]. Multiple sequence alignments of characterized genes and related amino acid sequences from MAGs in this study were created with Clustal-Omega v1.2.4 [60]. Each alignment was manually inspected for conserved active site residues essential for structure and function in AliView v1.28 [61]. HMMs were created from the inspected multiple sequence alignments with “hmmbuild” in HMMER 3.3.2 [62]. The HMMs were first tested on MAGs from this study using “hmmsearch” and the trusted cutoff (TC) values were iteratively adjusted to ensure only genes with conserved residues were captured. The resulting HMMs were used to retrieve denitrification gene sequences from *Firmicutes* genomes (*Firmicutes* and *Firmicutes* A-H, *n* = 13 543) downloaded from GTDB R07-RS207 [63] using “gtt-get-accessions-from-GTDB” in GToTree v1.6.34 [64]. Note that *Firmicutes* phyla were renamed *Bacillota* following the release of GTDB reference data R214. Denitrification gene sequences from GTDB and thermospore MAGs were dereplicated with “fastx_uniques” in USEARCH v11 [65]. Unique sequences were aligned with Clustal-Omega v1.2.4 [60], manually inspected, and included in a revised HMM. TIGRfams were used to identify the genes *narGH*, *napA*, *nosZI* (clade I), and *nosZII* (clade II) [66]. Following manual inspection, the TC for the *napA* HMM was amended to capture monomeric NapA found in *Bacillota* E genomes. The final HMM set is available at https://github.com/emma-bell/metabolism.

### Visualization of denitrification genes in *Bacillota*

*Bacillota* genomes from GTDB with Nor and/or Nos genes identified in their genome (*n* = 433) were visualized in a phylogenomic tree with thermospore MAGs from this study. The tree was created with GToTree v1.6.34 [64] from a concatenated alignment of 119 single copy genes targeted by the *Firmicutes* HMM profile. Within GToTree, genes were first predicted with Prodigal v2.6.3 [67] and target genes were identified with HMMER3 v3.3.2 [62]. Target genes were individually aligned with muscle v5.1 [68] and trimmed with TrimAl v1.4.rev15 [69]. Concatenated sequence alignments were used to create a maximum-likelihood phylogenomic tree using the Jones-Taylor-Thornton substitution model in FastTree2 v2.1.11 [70]. An *Actinobacteriota* MAG from this study was used as outgroup to root the phylogenomic tree. The tree was transformed and annotated in Treeviewer v2.2.0 [71].

## Results

### Enrichment of thermophilic denitrifiers

**Figure 1:**
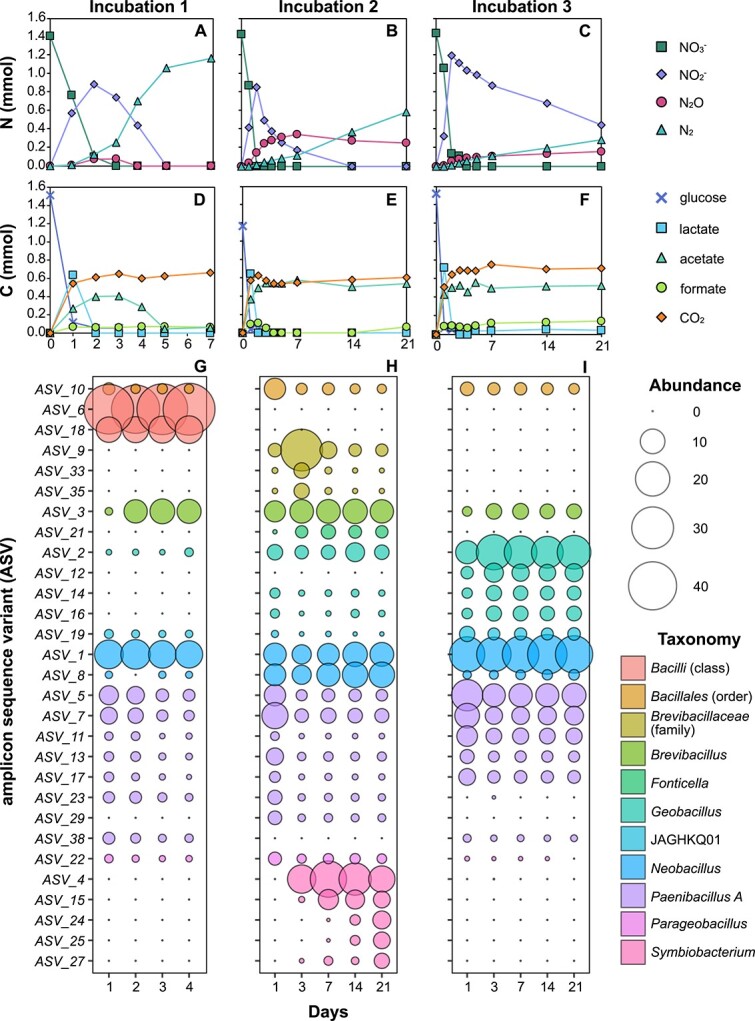
**Thermospores enriched in heated nitrate-amended incubations.** Nitrate reduction and production of nitrite, nitrous oxide and dinitrogen was monitored during incubation at 50°C (**A**, **B**, **C**) (nitric oxide was not measured). Glucose, organic acids, and CO_2_ measurements for each incubation are shown in corresponding panels underneath (**D**, **E**, **F**). All incubations were monitored over 21-days (Table S5) and measurements are shown for either 7-days (**A**) or 21-days (**B, C**) for clarity of nitrogen transformations. 16S rRNA gene amplicons were sequenced from multiple select time points (**G**, **H**, **I**) and *Bacillota* represented 94–98% read abundance in all cases. Only ASVs detected at >2% read abundance are included in the plots.

**Figure 2:**
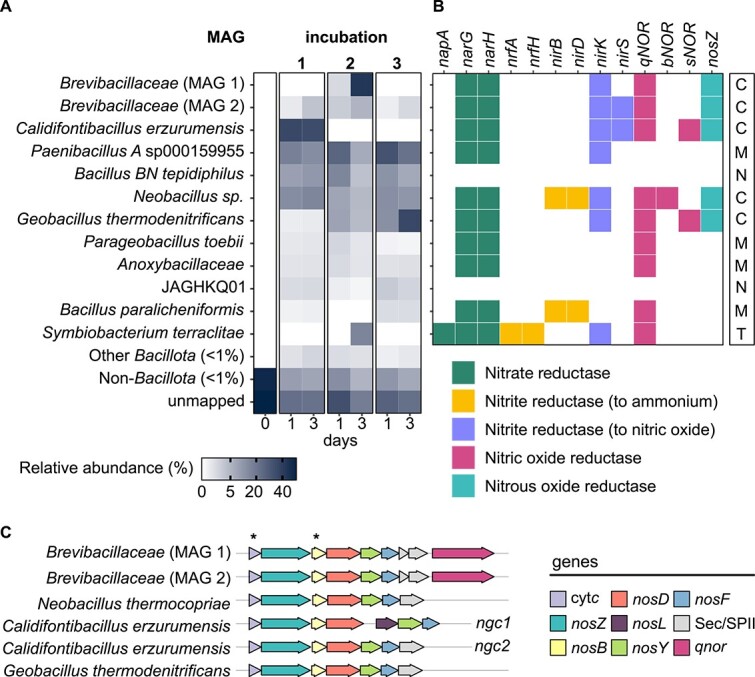
**Denitrification genotypes of enriched thermospores.** Relative abundance of thermospore MAGs in incubations with NO_3_^−^ after one- and three-days incubation at 50°C **(A)**. For comparison, the abundance of thermospores in the inoculum, prior to their enrichment, is shown as 0 days. Based on denitrification genes present **(B)**, MAGs were classified as complete (C), truncated (T), modular (M) or non-denitrifying (N) with respect to their potential for denitrification (column at right). Complete denitrifiers encode clade II-type NosZ. The Nos gene cluster (ngc) of thermospore MAGs is shown **(C)** including two gene clusters in the single *Calidifontibacillus erzurumensis* MAG. Asterisks indicate conserved genes found in clade II.

Incubation of oil sands at 50°C with nitrate and glucose resulted in nitrogen compounds being sequentially reduced (Fig. 1A–C) coupled to the metabolism of glucose into organic acids and carbon dioxide (Fig. 1D–F). Gas production was variable between replicates, with ~88, 59, and 31% of added N-NO_3_^−^ accounted for in the gas phase (i.e., combined N-N_2_O and N-N_2_; see Table S5) of the three incubations. This suggests ammonium was also produced by nitrate metabolism. Organic acid consumption was also variable. Consistent with these observations, 16S rRNA gene amplicon sequencing showed that distinct populations were enriched in different bottles (Fig. 1G–I). Amplicon sequence variants (ASVs) from the genera *Brevibacillus*, *Neobacillus*, *Geobacillus*, *Paenibacillus*, and JAGHKQ01 (family *Bacillaceae G*) were found in common across triplicate enrichments, whereas other taxonomic lineages were exclusive. For example, ASVs of the class *Bacilli* (ASVs 6 and 18) were enriched only in incubation 1 (Fig. 1G) whereas ASVs of the family *Brevibacillaceae* (ASVs 9, 33, 35) and genus *Symbiobacterium* (ASVs 4, 15, 24, 25, 27) were enriched only in incubation 2 (Fig. 2H). This experimental approach therefore showed potential to uncover a diverse range of thermospores, with different members of the oil sands microbial seed bank becoming enriched from within parallel inocula.

### Reconstruction of thermospore MAGs

Amplicon sequencing profiles showed the microbial communities to be consistent over time (Fig. 1G-I), which led to the selection of two time points from each incubation to more comprehensively survey the diversity of thermospores with metagenomic sequencing. In addition to sub-samples taken from the 50°C incubations after 1 and 3 days, metagenomic sequencing was performed on the inoculum (i.e., unheated oil sands). Twelve high quality non-redundant MAGs from *Bacillota* phyla (i.e., *Bacillota* ×11; *Bacillota* E × 1) were recovered from heated incubations (Fig. 2A). 16S rRNA gene sequences were present in MAGs of *Geobacillus thermodenitrificans* and *Brevibacillaceae* (MAG 2), which correspond to ASV_2 (99% identity) and ASV_3 (100% identity), respectively (Fig. 1G-I). Reads from the *Bacillota* MAGs were not detected in the unheated oil sands metagenome (Fig. 2A) which is consistent with dormant thermospore populations only germinating upon heating. Genomic potential for endospore formation and germination in *Bacillota* MAGs was also confirmed by the presence of functional and regulatory genes conserved within endospore-forming taxa (Table S3).

### Identifying genes for denitrification

KEGG orthologs (KO) did not capture the diversity of denitrification enzymes present in *Bacillota*. With KEGG, the Nor qNOR was annotated as subunit B of cNOR (*norB*, K04561) and Nors bNOR and sNOR are annotated as the related but functionally distinct cytochrome *c* oxidase (*coxA*, K02274). Nitrous oxide reductase is identified with KEGG (*nosZ*, K00376) but clade I and clade II enzymes are not differentiated. Furthermore, the only gene annotated in addition to *nosZ* from the Nos gene cluster was the accessory protein *nosD* (K07218), found in both clade I and clade II *nosZ* microorganisms [72]. A set of HMMs was therefore created to capture denitrification potential in genomes of *Bacillota*. The HMM set includes distinct HMMs for Nors and differentiates between clade I and clade II NosZ. An HMM for the membrane lipoprotein *nosB* is also included. NosB is essential for N_2_O respiration in clade II *nosZ* microorganisms but is commonly absent in clade I microorganisms [24, 53].

Using the HMM set, MAGs were designated as complete-, truncated-, modular-, or non-denitrifiers based on genes present (Fig. 2B). A MAG was designated complete if genes for each step of the denitrification pathway are present. A MAG was called truncated if the genome lacked only *nosZ*, suggesting the end product of nitrate metabolism is N_2_O rather than N_2_. A MAG was considered modular if it lacked *nosZ* in addition to any other genes from the denitrification pathway, suggesting it can only participate in certain reductive steps. Finally, a MAG was called non-denitrifying if it contains no genes for reductive N metabolism.

### Genomic potential for complete denitrification in thermospores

Potential for complete denitrification was found in five thermospore MAGs (Fig. 2B). Each of the five MAGs contain a membrane-associated *nosZ* with Sec-type signal peptide characteristic of clade II enzymes [23] and all enzymes in the pathway were predicted to be located in the cytoplasmic membrane, as expected for gram-positive denitrifiers [73]. Complete denitrifiers are taxonomically classified as *Calidifontibacillus erzurumensis*, *G. thermodenitrificans*, *Neobacillus* sp., and novel members of the family *Brevibacillaceae* (×2). The two *Brevibacillaceae* MAGs shared just 71.4% AAI with each other and comparison to members of this family in GTDB revealed no close relatives (>70% AAI). The greatest AAI was shared with an uncharacterized thermophilic soil bacterium, *Brevibacillaceae* species CFH-S0501 sp011059135, at 68.4 and 70.4% AAI, respectively.

Three Nor genes (*qnor*, *bnor*, and *snor*) were present in thermospore MAGs. The genes *bnor* and *snor* were found in genomes of complete denitrifiers in addition to *qnor* (Fig. 2B). Two complete denitrifiers, *Calidifontibacillus erzurumensis* and *Brevibacillaeace* (MAG 2), contained two nitric oxide-producing Nirs with both a *nirS* and a *nirK* gene. In addition to nitric oxide-producing *nirK*, *Neobacillus* sp. also contained an ammonium-producing Nir (*nirBD*) that can support both assimilation and dissimilation [74].

Clade II denitrifiers have a Nos gene cluster that differs to clade I denitrifiers, featuring some conserved genes that are absent in clade I genomes [24, 72]. Assessment of the Nos gene cluster (Fig. 2C) showed a cytochrome *c* preceding *nosZ* in all five thermospore genomes, as found in other clade II microoorganisms [54]. A gene with four transmembrane helices characteristic of *nosB* was adjacent to the putative ABC transporter complex *nosD, -Y, -F* in all of those genomes. *Calidifontibacillus erzumensis* had two Nos gene clusters, one of which had the copper chaperone *nosL* but this gene was absent from the genomes of other thermospores. The Nos gene cluster of *Brevibacillaceae* also differed from the other thermospores in that it contained a Nor (qNOR) immediately adjacent to the Nos gene cluster, whereas this gene was found elsewhere in the genome in the other three thermospore genomes.

### Truncated or modular denitrification potential in thermospores

Genes from the denitrification pathway were detected in five *nosZ*-lacking thermospore MAGs. These thermospores were designated truncated or modular in their metabolic potential for denitrification (Fig. 2B). *Symbiobacterium terraclitae* was the only MAG designated as truncated and was the only MAG to contain both *nar* and *nap* nitrate reductases (Fig. 2B). The *nap* nitrate reductase in *S. terraclitae* is monomeric (*napA*) and is distinct from the heterodimeric *napAB* commonly found in Gram-negative bacteria [75]. Ammonium-producing nitrite reductase (*nrfAH*) was present in this MAG, suggesting *S. terraclitae* can also perform DNRA. Production of NH_4_^+^ by *S. terraclitae* could account for the proportion of added N-NO_3_^−^ that is unaccounted for in the gas phase of incubations featuring germination and enrichment of this thermospore (Fig. 1B and Table S5).

Ammonium-producing Nir (*nirBD*) was also present in *Bacillus paralichenformis* (Fig. 2B), which was present in low relative abundance in incubations 1 and 3 (Fig. 2A). The *B. paralichenformis* MAG also contained Nor (*qnor*). In isolates of this species *qnor* is reported to play a role in detoxification of nitrite during DNRA resulting in the concomitant production of nonstoichiometric N_2_O [74, 76]. DNRA metabolism by *B. paralichenformis* could therefore contribute to both NH_4_^+^ and N_2_O production. Other MAGs designated modular have in common a respiratory nitrate reductase (membrane-bound *nar*), quinol-dependent nitric oxide reduction (*qnor*) and/or Cu-type nitrite reductase (*nirK*).

### Non-denitrifying thermospores

Two thermospore MAGs from the denitrifying enrichments contain no genes for respiratory nitrate metabolism. *Bacillus BN tepidiphilus* reached >10% relative abundance within one day of incubation and JAGHKQ01 (family *Bacillaceae G*) maintained a comparatively lower abundance (<2.5%) in all enrichments (Fig. 2A). Both of these genomes encode potential for glucose metabolism (mixed acid fermentation, sugar transport) indicating that they became enriched by fermentative growth. Populations that ferment sugars likely provided substrates to nitrate-reducing populations in the form of fermentation products such as lactate, acetate and formate that were observed to increase in the early hours of 50°C incubations (Fig. 1D–F).

### Denitrification genotypes of *Bacillota*

Representative genomes from *Bacillota* phyla (*Bacillota* and *Bacillota* A-H) were retrieved from GTDB and screened for nitric oxide (cNOR, qNOR, bNOR, sNOR) and Nos (NosZI, NosZII). Nitric oxide and/or Nos genes were present in ~10% of *Bacillota* genomes (*n* = 392/4216), ~5% of *Bacillota C* genomes (*n* = 20/395), ~4% of *Bacillota B* genomes (n = 12/323), and ~ 1.5% of *Bacillota E* genomes (*n* = 5/65). Just four genomes from *Bacillota A* (*n* = 8243) and *Bacillota G* (*n* = 131) contained either gene and all genomes from the phyla *Bacillota D*, *F*, and *H* (170 genomes) lacked both.

**Figure 3:**
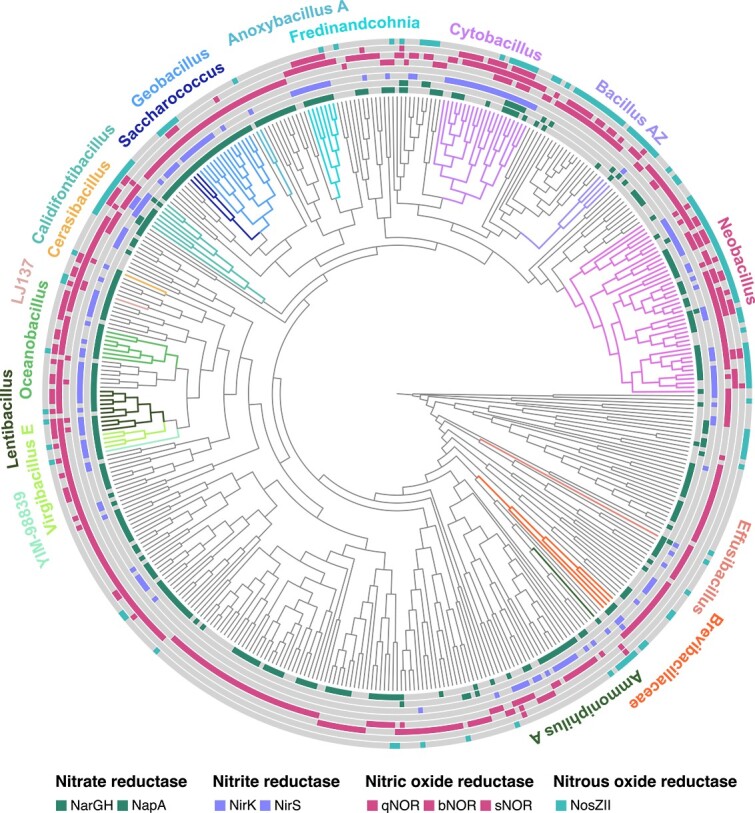
***Bacillota* genera with genomic potential for complete denitrification.** A phylogenomic tree was constructed with *Bacillota* MAGs from this study and 433 genomes from GTDB that contain nitric oxide reductase and/or Nos. Colored wedges at the tips of branches indicate gene presence (filled) or absence (grey). Genes shown from the innermost circle to the outer: *narGH*, *napA*, *nirK*, *nirS*, *qnor, bnor, snor,* clade II-type *nosZ*. The phylogenomic tree was constructed using a concatenated alignment of 119 single copy genes conserved within *Bacillota* and is presented as a cladogram. Within the phylum *Bacillota*, 17 genera have potential for complete denitrification (colored clades with bold text).

Genomes from *Bacillota* phyla with nitric oxide and/or Nos (*n* = 433) were screened with the full denitrification HMM set and included in a phylogenomic tree with MAGs from this study (Fig. 3). Genomic potential for complete denitrification is constrained to the phylum *Bacillota* and was only found within members of the class *Bacilli*. Seventeen genera contain complete denitrifiers (Fig. 3; 41 GTDB MAGs +5 MAGs from this study). This includes 11 genomes that encode bNOR and/or sNOR but lack qNOR (or cNOR) and would have been considered incomplete denitrifiers using KO annotations only.

Among denitrification genotypes, the absence of one or more of the reduction steps is common. A further 74 MAGs were categorized as truncated denitrifiers i.e., missing only Nos, 73 were categorized as nitric oxide reducers i.e., contain Nor only, and 15 were categorized as non-denitrifying nitrous oxide reducers i.e., contain Nos only (Fig. 3 and Table S6). The remaining genomes were categorized as modular i.e., they contain Nor and/or Nos in addition to one or more denitrification pathway genes. Many genomes from *Bacillota* phyla encode qNOR (172/433) or bNOR (123/433) whereas sNOR was rarely found without the presence of another Nor gene (2/433). Cytochrome *c*-dependent Nor (cNOR) was only encoded in genomes within the family *Desulfitobacteriaceae* from the phylum *Bacillota* B (6/433). *Desulfitobacteriaceae* also contain clade II-type *nosZ* but lack genes for nitrite reduction. Occurrences of three NOR genes in the same genome (qNOR, bNOR, and sNOR) was observed in 26 genomes, most of which belong to *Neobacillus* and other genera containing complete denitrifiers (Fig. 3).

## Discussion

Targeted enrichment with nitrate resulted in the germination and activity of denitrifying thermospore populations. This approach uncovered multiple lineages of denitrifiers, including novel members of the family *Brevibacillaceae.* Different thermospore taxa responded in parallel incubations suggesting there are heterogeneous populations of dormant thermospores in Athabasca oil sands outcrops, which is consistent with similar observations of oil sands microbiomes generally [29, 77]. It is well documented that thermospores comprise part of the microbial seedbank in cold sedimentary [78, 79] and soil environments [80, 81]. Germination can be activated by sublethal heat shock and addition of nutrients [82] with enrichment of different thermospore populations being influenced by both temperature and available substrates [78, 83, 84]. When conditions change favorably, dormant populations germinate and become active members of the microbial community. This premise underpins strategies for engineered microbial activity in situ with the objective of pressure generation and maintenance via microbial biogas such as N_2_ [27]. Our results demonstrate the feasibility for denitrifying thermospores to be stimulated in oil sands.

Biogas production was variable between replicates and can be attributed to the enrichment of distinct thermospore populations in different incubations. Genomic analysis showed potential for both denitrification and DNRA in different individual genomes as well as within the same genome, a feature that is not uncommon among *Bacillus* spp. [54, 85]. Co-occurrence of both pathways in a single genome highlights the challenge associated with predicting metabolic end-products based on gene content and the importance of environmental factors for determining metabolic activity [86, 87]. Metabolic end-products can also be influenced by the accumulation of intermediate metabolites. The protonated form of nitrite (HNO_2_) is toxic at high concentration [88] and could have influenced the germination and enrichment of thermospores detected in this study. Certain *Bacillus* spp. capable of DNRA have a high tolerance to nitrite, but detoxification results in a greater production of N_2_O [74, 76]. Nitrite has also been shown to inhibit N_2_O reduction during denitrification [89] resulting in reduced production of N_2_.

Denitrifying thermospores enriched from oil sands have clade II-type *nosZ* genes (subclade H) for catalyzing reduction of N_2_O to N_2_. Clade II *nosZ*-bearing microorganisms are numerically significant in the environment [90–93], though they are often considered to be non-denitrifiers lacking genes needed for the stepwise reduction of nitrate to dinitrogen [24, 94]. While this is true for certain lineages within the diverse clade II-type NosZ, analysis of MAGs from N_2_-producing enrichments in this study, as well as *Bacillota* from GTDB, shows that multiple genera within the class *Bacilli* contain a full complement of denitrification genes. This is consistent with multiple isolated representatives from this class that have been experimentally shown to perform complete denitrification [25]. Examples include *G. thermodenitrificans* isolated from a deep oil reservoir [95] and *Calidifontibacillus azotoformans* (formerly *Bacillus azotoformans*) isolated from soil [73].

Genomic potential for complete denitrification was present in 17 genera within the class *Bacilli*. Within these genera, it was common for microorganisms to possess multiple Nors (qNOR, bNOR, and sNOR). This highlights that NOR enzymes are not mutually exclusive, though the different conditions under which they are not expressed in *Bacillota* are not clear. It has been suggested that bNOR in *B. azotoformans* can be used for aerobic NO reduction in microoxic environments [54]. The recently characterized enzyme eNOR, that descends from the same family of oxygen reductases as bNOR, also reduces nitric oxide under microoxic conditions [22, 96]. Members of the *Bacillota* are often identified as contributors to denitrification in environments with variable redox conditions, including agricultural soil, deep vadose zone soil, and rice paddy soil [8, 97, 98] and can be present in the soil microbiome generally [99]. Having multiple Nors could provide *Bacillota* with metabolic versatility in environments like soils, where combined oxic and anoxic conditions are commonly found [4].

So-called functional redundancy has also been found with other enzymes within the denitrification pathway. *B. azotoformans* contains five Nos gene clusters, three of which include a *nosZ* gene (Heylen 2012). In addition, a recent survey of nitrite reductases (NirK and NirS) in isolates and MAGs showed that possessing both enzymes is more common than previously appreciated and potentially allows microorganisms bearing both enzymes to denitrify across a wider range of environments [100]. Functionally redundant enzymes within a genome may also reflect an ability of the microorganisms to adapt to changing environmental conditions. This would be a beneficial trait for members of the *Bacillota* as endospore-formers undergo periods of dormancy and respond rapidly through germination to changes in their environment.

Genome-resolved metagenomics is a useful approach for studying denitrification as it provides the gene content of populations and circumvents challenges with PCR-based marker gene approaches. However, we found that certain denitrification genes were missed, or pathways appeared incomplete, using standard annotation databases that are biased towards clade I denitrifiers. For example, the denitrification reference pathway in KEGG includes nitrate reductase composed of subunits *napAH* and Nor composed of subunits *norBC*. However, nitrate reductases in *Symbiobacterium* (*Bacillota E*) are monomeric [75] and lack the *napH* subunit. Similarly, qNOR Nors are fused and lack the *norC* subunit [18]. This can result in modules or pathways appearing incomplete. To date bNOR has only been found in *Bacillota* and was originally isolated from *B. azotoformans* [21, 73]. Despite being biochemically characterized bNOR genes were not identified with commonly used gene annotation databases KEGG, eggNOG, or TIGRfam. Finally, while putative NOR enzyme families that have been recently proposed [22, 96] are not expected to be represented in curated annotation databases, their absence nevertheless highlights that interpretation of community gene content is limited by the breadth of gene databases. Considering the complete diversity of NOR enzymes reveals a greater diversity of microorganisms capable of denitrification. This is an important consideration for studies attempting to quantify capacity for denitrification or N_2_O emissions based on gene content in both natural environments and engineered systems to ensure that metabolic potential is not underestimated.

## Supplementary Material

Supplementary_Data_Tables_S1-S6_ycae107

## Data Availability

DNA sequencing data (16S rRNA gene amplicon, metagenome, and metagenome-assembled genomes) are available at the NCBI Sequence Read Archive under BioProject ID PRJNA1110647.
